# Future soil erosion trends in Canadian agricultural lands from runoff and sustainability impacts

**DOI:** 10.1038/s41598-025-05947-5

**Published:** 2025-07-02

**Authors:** Afshin Amiri, Isa Ebtehaj, Keyvan Soltani, Silvio Jose Gumiere, Hossein Bonakdari

**Affiliations:** 1 Department of Soils and Agri-Food Engineering, Université Laval, Quebec City, Québec G1V0A6 Canada; 2 Department of Civil Engineering, University of Ottawa, 161 Louis Pasteur Private, Ottawa, ON K1N 6N5 Canada

**Keywords:** Remote sensing, Soil erosion, USLE, Deep learning, CMIP6, Agricultural land, Hydrology, Natural hazards

## Abstract

Human activities have significantly altered agricultural regions, leading to critical issues such as reduced soil fertility, biodiversity loss, and accelerated soil erosion. Despite their importance, reliable erosion maps for Canadian croplands remain scarce, hindering effective mitigation strategies. Here, we aimed to map erosion-prone areas in Canada by combining remote sensing and artificial intelligence methods under current and future climate scenarios from the Coupled Model Intercomparison Project Phase 6 (CMIP6). Our results revealed that, on a national average, soil erosion in Canada ranges from 4.72 to 6.64 t/ha/yr. All the scenarios indicate an increase in soil erosion over time. Soil degradation could become a more severe problem in the future. Our findings revealed that by 2030, 81,038 km² of agricultural land will experience high and severe erosion risks, indicating a significant 53.9% increase compared with that in 2020. The development of accurate soil erosion risk maps will not only enhance targeted conservation efforts but also serve as a critical tool for policymakers to implement effective soil management strategies, contributing to sustainable agriculture and climate resilience at a broader scale.

**Supplementary Information:**

The online version contains supplementary material available at 10.1038/s41598-025-05947-5.

Fertile soil is one of the most important resources for sustainable development^1^. Natural geomorphological processes such as soil erosion (SE) have remained persistent worldwide for decades, particularly in Canada. The natural soil formation rate is lower than the soil erosion rate^2,3^. An imbalance between soil formation and erosion leads to significant risks for food security and ecosystem sustainability^3^. Although several nations are already making efforts to address this issue, worldwide soil erosion is continuously increasing^4^. Our agricultural systems are damaging the environment on a global scale^5–7^. Future food security and sustainability demands need to be addressed through an increase in food production and a decrease in agricultural environmental impacts^5^.

Soil erosion models can assist in land management by facilitating the identification of erosion-prone regions, the estimation of erosion rates, and the identification of potential causes of erosion^8^. The universal soil loss equation (USLE) was developed by the United States Department of Agriculture and Soil Conservation Services (USDA-SCS) to model the long-term average yearly soil loss^9,10^. The USLE is frequently employed to calculate soil loss^11–15^. The USLE can be used to estimate the yearly soil loss related to sheet and rill erosion processes via six factors: rainfall-runoff erosivity (R factor), soil erodibility (K factor), slope length and steepness (LS factor), land cover and management (C factor) and support practices (P factor). Among these soil erosion risk factors, the R, C, and P factors exhibit the greatest dynamic changes throughout the year^16^. The R factor is most important because precipitation directly affects aggregate separation and runoff and is the primary source of erosion^11,17^. Rainfall erosivity refers to the ability of rainfall to contribute to soil erosion on hillslopes via water^17^. Erosivity was described as a multiple of the rainfall kinetic energy and the maximum 30-minute intensity during individual storm events^18^.

Erosivity plays a crucial role in soil erosion risk assessment under future land use and climate change conditions. Several studies have assessed rainfall erosivity on monthly and annual time scales^16,19,20^. Detailed mapping of the intra-annual rainfall erosivity across Europe has demonstrated that the average rainfall erosivity in summer is approximately four times greater than that in winter^16^. A study involving a global potential soil erosion model with a fine resolution of 250 m that integrates remote sensing, geographic information system (GIS) modeling, and census data revealed the likelihood of an increasing global soil erosion issue, which is driven primarily by the widespread expansion of agricultural crops^4^. Some models estimate the average increase in global rainfall erosivity, which is driven primarily by climate change and increased rainfall erosivity^21^. A recent study that used the universal soil loss equation (RUSLE) estimated global and North American water and soil erosion rates under current and future climatic scenarios ranging from 12.3 to 15.8 and 11 to 15.2 t/ha/year, respectively^22^. Other studies have projected rainfall erosivity via different climate change scenarios from the Coupled Model Intercomparison Project (CMIP), revealing future increases in rainfall erosivity^23,24^.

Soil erosion represents a significant challenge in the realms of agriculture and natural resource management, posing serious threats to crop productivity and ecosystem sustainability. Although various studies have been conducted in Canada, a comprehensive assessment of soil erosion at the national scale utilizing the Universal Soil Loss Equation (USLE), particularly for agricultural lands, has yet to be undertaken. Addressing this gap is of paramount importance, as the degradation of soil in agricultural regions can lead to diminished fertility and compromised soil quality. Given the extensive area of agricultural land in Canada, remote sensing technology is a potent tool for estimating average soil erosion and analyzing prospective trends across diverse regions. This study seeks to bridge this research gap by providing critical data that can inform and enhance the management and conservation of soil resources. The USLE effectively incorporates critical factors such as rainfall intensity, soil type, land cover, and management practices, making it suitable for assessing soil erosion dynamics in Canadian agricultural lands. Additionally, it has been recommended by the Ontario Ministry of Agriculture, Food and Rural Affairs as a reliable method for assessing soil erosion. Given the lack of comprehensive studies using the RUSLE at the national scale in Canada, our research represents a pioneering effort.

Here, we provide a methodological framework that can be used to predict soil erosion variations in Canada by employing remote sensing, deep learning, and climate change models. To determine future soil erosion changes in Canada, the Canadian Earth System Model version 5 (CanESM5) model was analyzed. The main objectives of this research are to: assess historical soil erosion during the 2009–2020 period, predict future rainfall-runoff erosivity (R factor) from 2024 to 2100 using a Long Short-Term Memory (LSTM) deep learning approach, estimate future soil erosion through the USLE (assuming all factors remain constant except for the R factor), and identify high-risk erosion areas to inform soil conservation strategies and guide land management under changing climatic conditions.

This integrated approach not only fills a major gap in large-scale soil erosion modeling in Canada but also provides actionable insights for policymakers aiming to mitigate future land degradation under evolving climate scenarios. Notably, it applies the USLE model at the national scale in Canadian croplands—marking its first large-scale implementation in this context.

## Methods

### Study area

**Fig. 1:**
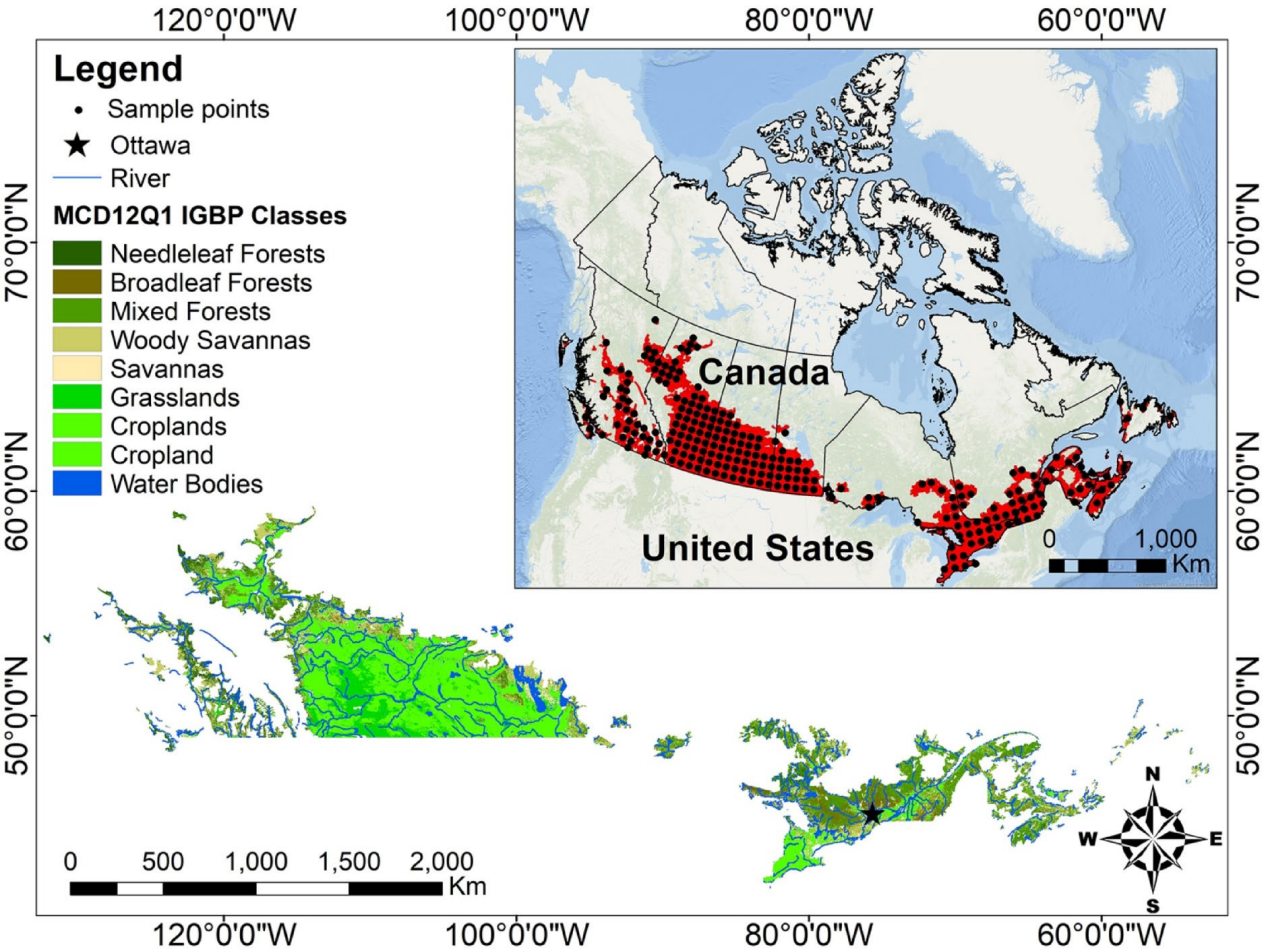
Map of the study area, including the Canadian agricultural landscape. The MODIS land cover type (MCD12Q1) version 6.1 data product shows the main land cover types in 2021. The distribution of 244 sample points in the study area is shown in the upper panel. The map was generated using ArcMap (version 10.8.2), available at https://desktop.arcgis.com.

The study area is restricted to parts of Canada with a land area of approximately 3.4 million km^2^ from approximately 42°N to 59°N latitude and 132°W to 52°W longitude, including ten provinces and territories (Fig. 1). The climate in Canada varies from cool temperate to arctic cold climates, with oceanic climates prevailing in coastal areas. The study area features temperature and precipitation gradients, with both increasing in the southern region^25^. Coastal regions are often warmer and exhibit longer growing seasons than the very seasonal northern regions of Canada, which exhibit heavy winter snowfall and brief summers. The climate in the central areas of Canada is continental, with long, cold winters and hot summers^26^. The average elevation of the study area is 535 m above mean sea level, and the maximum elevation is 2,919 m. The average annual precipitation in the study area is 849.8 mm on the basis of the Climate Hazards Group InfraRed Precipitation with Station dataset (CHIRPS) quasiglobal rainfall dataset^27^. According to the International Geosphere Biosphere Programme scheme land cover classification of the Moderate Resolution Imaging Spectroradiometer (MODIS) MCD12Q1^28,29^, the main land cover types in the study area are croplands, which account for 35.6% of the total area; mixed forests, which account for 17.8% of the total area; woody savannas, which account for 16.9% of the total area; and grasslands, which account for approximately 12.4% of the total area.

### USLE model

The universal soil loss equation (USLE) is a widely known soil erosion estimation model where the mass of soil lost per unit area and time (t/ha/yr) due to interrill and rill erosion processes can be obtained via the multiplication of five contributing factors^9,10^. Soil displacement due to processes such as gullying and tillage erosion is not estimated. To predict soil erosion, the determinants are first characterized and then combined via Eq. (1)^10^:

1 $$E=R \times K \times LS \times P \times C$$

where E is the estimated annual soil erosion per hectare (t/ha/yr), R is the rainfall-runoff erosivity (MJ mm h^− 1^ ha^− 1^ yr^− 1^), K is the soil erodibility (Mg h MJ^− 1^ mm^− 1^), LS is a parameter capturing the effect of the topography (dimensionless), C is a factor that captures the effect of land cover (dimensionless), and P is the support practice factor (dimensionless). The USLE variables were mapped at a 25 km resolution to ensure consistency across all the input datasets. However, the original datasets varied in spatial resolution. Higher-resolution datasets, such as 500 m MODIS-based and 30 m Landsat-based data, were resampled via the nearest neighbor method to preserve class integrity. This method ensures that each 25 km pixel reflects the dominant class from the underlying higher resolution pixels, preventing blending or artificial smoothing of class boundaries.

#### Rainfall-runoff erosivity (R)

One of the most essential input factors for defining erosive processes is rainfall-runoff erosivity. The rainfall-runoff erosivity factor reflects the overall energy of water droplets, which significantly impacts soil aggregate stability and accelerates the soil erosion process. Since soil erosion is difficult to quantify across broad regions, models are needed to estimate soil loss due to water erosion at regional and global scales^31^. The R factor from the global precipitation measurement (GPM)^32^ can be obtained via Eq. (2) ^10^:

2 $$R=\frac{1}{n}\sum\limits_{{j=1}}^{n} {\sum\limits_{{k=1}}^{{{m_j}}} {{{(E{I_{30}})}_k}} }$$

where EI_30_ is the rainfall-runoff erosivity during a single event (J.m^− 2^.mm.h^− 1^), n is the number of years observed, m_j_ is the number of erosive events during a given year j, and k is the index of a single event with a corresponding erosivity EI_30_. The EI_30_ during a single event can be calculated via Eq. (3) ^10^:

3 $$E{I_{30}}=\sum {(KE \times d)} \times {I_{30}}$$

where KE is the kinetic energy (MJ ha^− 1^ mm^− 1^), d is the rainfall level (mm), and I_30_ is the maximum intensity recorded within 30 min during a rainfall event (mm.h^− 1^). KE can be calculated according to the rainfall intensity via Eq. (4) provided in Ref^10^. which is converted into metric units, as noted in Ref^33^. :

4 $$KE=0.1187+0.0873\log (i)$$

where i is the rainfall intensity (mm.h^− 1^).

#### Soil erodibility (K)

Soil erodibility reflects the vulnerability of the soil structure to scattering by water droplets and runoff transport. The K factor captures soil susceptibility to erosion and can be estimated via the soil organic matter content, soil texture, permeability and soil structure. The K factor was defined according to the methodology proposed in Ref^10^. as follows:

5 $$K=\frac{{(2.1 \times {{10}^{ - 4}}{M^{1.14}}(12 - OM)+3.25(s - 2)+2.5(p - 3))}}{{100}} \times 0.137$$

where M is the content of the silt plus fine sand fraction multiplied by 100 minus the clay fraction, OM is the organic matter content (percentage), s is the soil structure, and p is the permeability class.

The current study used the K factor data of Borrelli et al.^4^who employed a combination of remote sensing and statistical data to model soil erosion patterns on a global scale. This dataset has a 25 km resolution and was developed at a global scale. These data are available at (https://esdac.jrc.ec.europa.eu/content/global-soil-erosion).

#### Slope r (LS)

The LS factor reflects the effect of the terrain on surface runoff and the capacity for sediment transportation. This factor was calculated via the GIS-based two-dimensional terrain approach (Eq. (6)) proposed in Ref^34^. :

6 $$LS=\frac{{{{({A_{i,j - in}}+{D^2})}^{m+1}} - A_{{i,j - in}}^{{m+1}}}}{{{D^{m+2}} \times x_{{i,j}}^{m} \times {{22.13}^m}}}$$

where A_i, j−in_ is the contributing area at the inlet of grid cell (i, j) (m^2^), D is the pixel size (m), x_i, j_ is calculated via Eq. (7), and m is related to the ratio β (Eq. (8)) of rill erosion to interrill erosion.

7 $${X_{i,j}}=\sin {a_{i,j}}+\cos {a_{i,j}}$$

where a_i, j_ is the aspect direction of grid cell (i, j).

8 $$m=\frac{\beta }{{\beta +1}}$$

9 $$\beta =\frac{{\frac{{\sin \theta }}{{0.0896}}}}{{[0.56+3 \times {{(\sin \theta )}^{0.8}}]}}$$

where θ is the slope angle. We used the LS factor data proposed by Borrelli et al.^4^who adopted a combination of remote sensing and statistical data to model soil erosion patterns on a global scale. This global dataset has a 25 km resolution and is available at https://esdac.jrc.ec.europa.eu/content/global-soil-erosion.

#### Conservation support practice factor (P)

The ratio of the soil displacement while applying a given conservation support practice, such as contouring, strip cropping, terracing, or subsurface drainage, can be expressed via the P factor (dimensionless). The primary data source for the P factor is the National Aeronautics and Space Administration (NASA)’s MODIS land cover type product (MCD12Q1), which is categorized according to the International Geosphere–Biosphere Programme (IGBP) scheme. The data were collected from NASA’s Earth Data site (https://reverb.echo.nasa.gov) and preprocessed via the Google Earth Engine (GEE) to generate baseline land cover maps. To transition from soil cover conditions to soil erosion impacts, values were allocated to groups of land cover types with comparable erosion effects. To calculate the P factor, values suggested by the Ministry of Agriculture, Food and Rural Affairs for Canada were used (Table 1). Since each pixel could contain multiple land cover types, a weighted average was used to represent the combined influence of different land covers. Nonagricultural areas were assigned a P factor of 1, reflecting the absence of soil conservation measures.

**Table 1:** P factor data for support cropland practices on the basis of the ministry of agriculture, food and rural affairs for canada.

Support practice	P factor
Up- and downslope	1.0
Cross slope	0.75
Contour farming	0.5
Strip cropping (cross slope)	0.37
Strip cropping (contour)	0.25

#### Land cover and management factor (C)

The C factor (dimensionless) captures the cumulative influence of interconnected soil cover and management factors on soil erosion. The annual crop inventory (ACI) layer was used to calculate the C factor. These data have a spatial resolution of 30 m and are produced via the decision tree (DT) method on the basis of optical and radar satellite images. This dataset is available from 2009 to 2023 and can be accessed at https://open.canada.ca/data/en/dataset/ba2645d5-4458-414d-b196-6303ac06c1c9. The C factor was calculated via the GEE by assigning a crop type value to each pixel, as suggested by the Ministry of Agriculture, Food and Rural Affairs for Canada (Table 2). The C factor offers comparable statistics for various cropping systems, assisting in evaluating the relative advantages of each system. For nonagricultural lands, such as forests, a C factor of 0.001 was assigned, as these areas contribute minimally to soil erosion.

**Table 2:** C factor per crop type on the basis of the ministry of agriculture, food and rural affairs for canada.

Crop type	C factor
Grain corn	0.4
Silage corn, beans and canola	0.5
Cereals (spring and winter)	0.35
Seasonal horticultural crops	0.5
Fruit trees	0.1
Hay and pasture	0.02

**Fig. 2:**
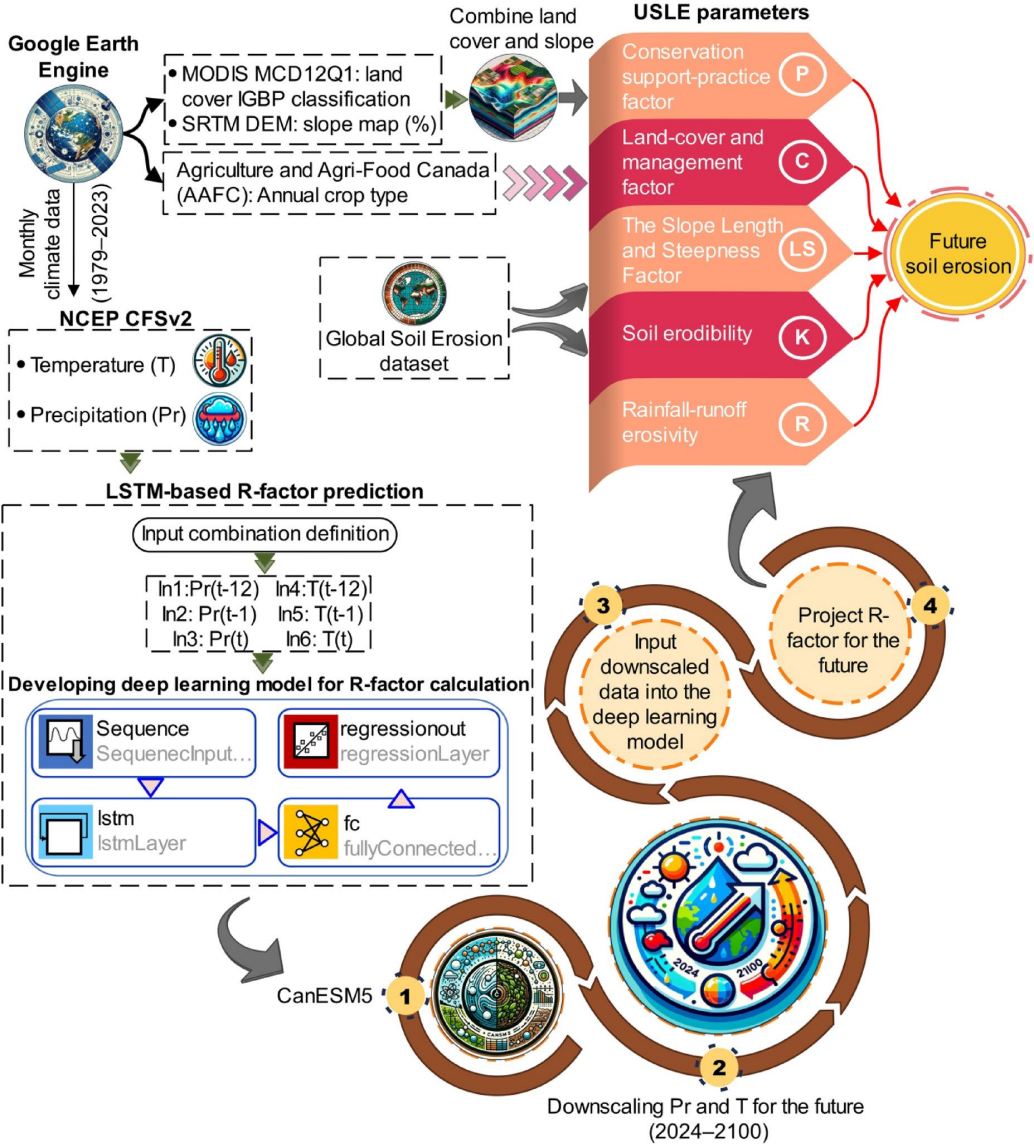
Flow chart of the soil erosion projection method. The P and C factors were calculated via Google Earth Engine, whereas the R factor was estimated via a deep learning model. Future precipitation (Pr) and temperature (T) data were downscaled via CanESM5 data, and the projected R factor was integrated with USLE parameters to estimate future soil erosion.

It is necessary to compute the parameters of the USLE model before the quantity of soil erosion can be estimated. The P and C factors were calculated via a JavaScript code developed in the GEE environment. Landcover and slope percentage maps were produced by processing the MCD12Q1 MODIS product and the Shuttle Radar Topography Mission (SRTM) DEM data, respectively. These maps were then combined according to Table 1 to obtain the P factor. To calculate the C factor, the main crop land cover types in the study area were extracted from the ACI dataset on the GEE platform and classified into six categories according to Table 2. In the short term, no notable changes in the LS and K factors exist. The global soil erosion dataset provides these two parameters^4^. The LS and K factors were masked at a 25 km resolution for Canada. In this study, by assuming that the C, P, LS, and K factors are constant and by predicting changes in the future R factor as the factor influencing erosion the most, the amount of erosion in the future in Canada was estimated. In this way, long-term time series data on precipitation and temperature (1979–2023) were obtained from the National Centers for Environmental Prediction (NCEP) Climate Forecast System version 2 (CFSv2) dataset for 244 sample points (Fig. 1). These data were employed to develop a deep learning model to calculate the R factor. The flow chart of this study is shown in Fig. 2. Unlike traditional statistical methods, the LSTM network was selected to predict future rainfall-runoff erosivity (R-factor) because of its superior capability to capture nonlinear and long-term temporal dependencies inherent in precipitation and temperature series. Given the complex interactions between temperature, precipitation, and rainfall erosivity, LSTM offers a robust approach for dynamically modeling these relationships over time, especially under diverse climate change scenarios^35,36^. Moreover, LSTM is particularly well-suited for modeling time series data with lagged effects, making it appropriate to project future erosivity based on historical climate patterns and CMIP6 climate projections. By applying LSTM, more accurate and flexible forecasting of the R-factor, which is considered a critical input for the USLE model used in soil erosion estimation, was aimed to be achieved.

The LSTM model was specifically employed to predict future R-factor values based on historical monthly temperature and precipitation data obtained from 244 sampling points across Canada. These R-factor projections were then integrated into the USLE framework to estimate future soil erosion rates, while keeping other USLE factors (K, LS, C, P) constant. This approach allows isolating and examining the influence of climate-induced erosivity changes on future soil erosion. The model uses past climate data as a learning set, enabling it to predict erosivity under various CMIP6 climate scenarios (SSP119, SSP126, SSP245, SSP370, SSP585). Thus, LSTM serves as the bridge between future climate projections and soil erosion modeling by dynamically estimating the most sensitive factor, R.

The LSTM model takes precipitation (Pr) and temperature (T) as inputs, each with multiple lag times. Specifically, both Pr and T are considered at three time steps: the current time (t), one step prior (t-1), and twelve steps prior (t-12). Consequently, six input features (i.e., Pr(t-12), Pr(t-1), Pr(t), T(t-12), T(t-1), and T(t)) were generated and provided to the LSTM network to model the monthly R-factor values dynamically. After the inputs are defined, they are fed into the LSTM model, which consists of four main layers in this study: a sequence input layer, an LSTM layer, a fully connected layer, and a regression layer. These layers were selected after numerous combinations of different architectures were evaluated. Additionally, the optimal LSTM hyperparameters were determined through a grid search process, yielding the following values: maximum number of epochs: 2000, mini-batch size: 120, gradient threshold: 0.05, initial learning rate: 0.005, learning rate schedule: ‘piecewise’, learning rate drop period: 125, and learning rate drop factor: 0.9. Moreover, the optimizer algorithm used was mean square propagation (RMSProp). RMSProp is an adaptive optimization algorithm that dynamically adjusts the learning rate for each parameter, stabilizing training compared with fixed-rate methods such as SGD. It scales the learning rate via the exponential moving average of squared gradients, preventing drastic updates and mitigating vanishing or exploding gradients. Designed for nonstationary problems, RMSProp excels in time series forecasting and sequence-based tasks by adapting to changing gradients, making it a preferred choice for training deep networks, including LSTMs.

### Deep learning model

Training recurrent or profoundly complex neural models can be challenging owing to the prevalent issue of gradients either increasing or decreasing exponentially^37^. To address this limitation in capturing extended data sequences, the introduction of the LSTM framework^38^ was pivotal. This advanced recurrent neural framework, i.e., LSTM^38^is tailored to navigate the challenges of vanishing and exploding gradients, ensuring efficient learning of extended sequences, even in scenarios with significantly long intervals^39^.

This issue can be addressed via a constant error carousel (CEC), which maintains the error signal within each unit’s cell. These cells function as recurrent networks that incorporate input and output gates to form a memory cell. The self-recurrent connections enable feedback with a one-time-step delay, enhancing error preservation and stability.

A basic LSTM unit comprises a cell, an input gate, an output gate, and a forget gate. The core component is the cell state, allowing information to be added or removed as needed. This process is controlled by gates, which regulate information flow via a sigmoid neural network layer and pointwise multiplication. The sigmoid layer outputs values between zero and one, describing how much information passes through. In an LSTM, there are three distinct types of gates that play crucial roles in its functioning. Among the three gates, the forget gate plays a key role in filtering out unnecessary data, deciding what information should be retained or discarded from the cell state. The inclusion of the forget gate, which was not part of the original LSTM design, was suggested in Ref^40^. to enable the network to clear its memory. The cell stores information for varying durations, whereas the three gates regulate its flow. The input gate updates the cell state by introducing new information, allowing the LSTM to adapt to changing inputs. The output gate determines the next hidden state, enabling the model to make predictions or on the basis of both past and new inputs. Together, these gates enable the LSTM to manage long-term dependencies, effectively capturing complex temporal patterns.

**Fig. 3:**
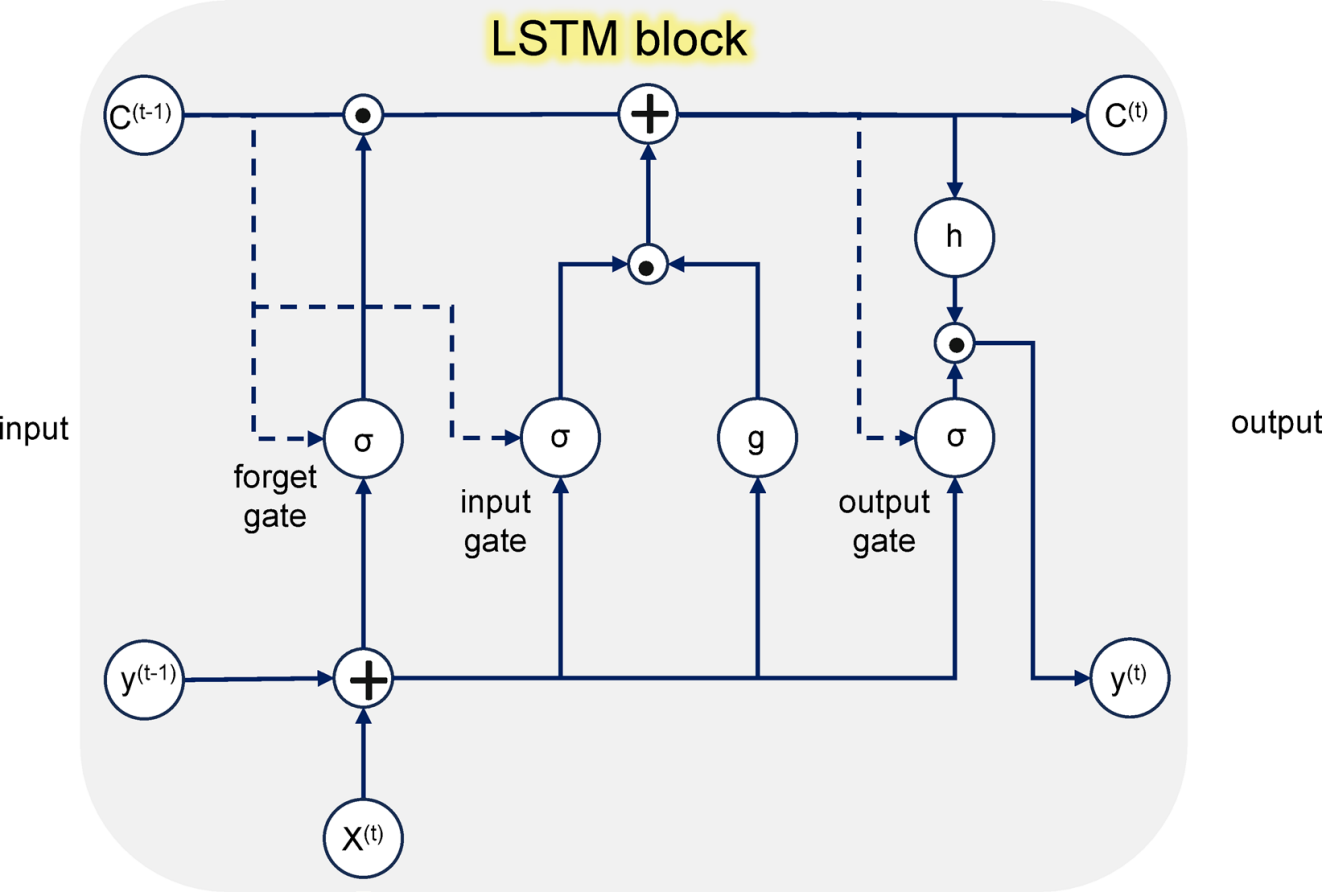
The architecture of the basic LSTM block. This model entails the use of a sequence of gates to regulate the flow of information, allowing it to remember and forget patterns over time selectively.

The design of a standard LSTM block is shown in Fig. 3, which shows the gates, the input signal x(t), the activation mechanisms, the peephole connections, and the output y(t)^40^. The block output is looped back into both the input and all gates of the block, thus creating a recurrent connection.

We developed a MATLAB script to implement the LSTM model for predicting the rainfall-runoff erosivity factor (R-factor) using lagged precipitation and temperature data combined with spatial coordinates. The implemented code is available in Supplementary Table 1. The script begins by loading meteorological and geographical data from Data.mat, which includes precipitation (Pr_obs), temperature (T_obs), spatial coordinates (Coordinate), and rainfall-runoff erosivity factor (R). These variables are then standardized using a custom Normalization function that performs min-max scaling, a crucial preprocessing step for stabilizing the training of neural networks. Specifically, minimum and maximum values of the R variable are stored to later reverse the normalization process and obtain predictions in their original scale.

To prepare the data for time series modeling, the script introduces a lag of 12-time steps, enabling the model to learn from one year’s worth of historical data. The TS function constructs lagged input matrices for each variable, where every row captures a sequence of the current and preceding 12 months. The model’s input features are constructed by selecting specific lags (1, 2, and 12) from both precipitation and temperature variables, and concatenating them with spatial coordinates. The output variable is defined as the final (current) value of the lagged R matrix, effectively setting up a supervised learning task to predict the R-factor based on the given spatiotemporal context.

A critical element of this workflow is the careful partitioning of the dataset into training (50%), validation (25%), and independent testing (25%) subsets. This partitioning ensures that the model is trained on one part of the data, tuned and evaluated on a separate validation set, and ultimately assessed on an entirely untouched test set. The independent test set thus provides an unbiased estimate of the model’s generalization performance. To robustly evaluate the model, a 10-fold cross-validation procedure is implemented over the combined training and validation set (75% of the data). This approach cyclically trains the model on 90% of that subset while validating on the remaining 10%, rotating through all possible folds. Each fold involves re-training the LSTM model using a deep neural architecture comprising a sequence input layer, a hidden LSTM layer with 100 memory units, a fully connected layer, and a regression layer for output. The training uses the RMSprop optimizer with a learning rate schedule and a maximum of 2,000 epochs. Validation data within each fold are used to monitor training and adjust the learning process accordingly.

After training in each fold, the model predicts values for the training set, the validation fold, and the previously held-out independent test set. All predictions are denormalized using the stored min and max values to interpret the results in real-world units. The script then calculates performance metrics—Root Mean Square Error (RMSE) and coefficient of determination (R²)—on the validation folds and aggregates the results across all folds to report mean performance. Finally, the code defines three supporting functions. The Normalization and Denormalization functions handle scaling transformations, ensuring consistency between training and inference phases. The TS function automates the generation of lagged time series matrices, a reusable component applicable to any temporal variable in the dataset.

LSTMs are highly effective at capturing long-term dependencies in sequence data, making them essential for tasks that require understanding context over extended. LSTMs are also flexible in handling inputs of varying lengths, making them suitable for diverse applications, from short texts to lengthy documents or time series data. This adaptability and ability to retain crucial information over time make them powerful tools for sequential data analysis.

### Climate data and climate change

The monthly temperature and precipitation data for the study area from 1979 to 2023 were extracted from CFSv2 6-hourly products with a resolution of 22.2 km^30^. The National Centers for Environmental Prediction (NCEP) provided this dataset. The temperature and precipitation data were acquired at 244 sample points across the study area (Fig. 1) and converted into monthly data via the GEE computation platform.

GPM-IMERG (Global Precipitation Measurement – Integrated Multsatellite Retrievals for GPM) is a satellite-based precipitation dataset that provides high-resolution and near-global precipitation estimates^32^. GPM-IMERG offers precipitation data at fine temporal (30-minute) and spatial (0.1°) resolutions, making it a valuable resource for hydrological and climatological studies. The dataset is particularly useful for assessing rainfall erosivity, which is a key component in estimating soil erosion rates.

The R factor was estimated via an LSTM model trained on historical monthly precipitation and temperature data. The trained model was then applied to projected climate data from five climate scenarios to estimate future R-factor values. This approach captures the nonlinear influence of precipitation and temperature variations on rainfall erosivity, ensuring consistency between historical and future estimations.

We utilized the CanESM5 model, which is based on the latest Intergovernmental Panel on Climate Change (IPCC) report (CMIP6) (https://www.ipcc.ch/assessment-report/ar6/), to estimate precipitation and temperature values. Developed specifically for Canada, the CanESM5 model is the main choice for deriving climatic parameters tailored to the Canadian context. To explore various climate scenarios, we employed five scenarios: SSP126, SSP245, SSP370, SSP585, and SSP119. These SSPs represent different socioeconomic development pathways and provide a range of possible future climate conditions. We adopted a change factor method (CFM)^41,42^also known as the delta method, in the downscaling process. The CFM is a statistical technique used to downscale climate model outputs by aligning them with historical observations. This method is particularly effective, as it allows for the integration of historical climate data to refine model outputs, ensuring that future projections are more relevant and contextually accurate. In the case of precipitation, the change factor is computed as the ratio of future to past monthly mean values. The temperature is derived from the difference between the future and historical monthly averages. This technique involves applying adjustments or scaling factors to the coarse-resolution output of the CanESM5 model to better conform with the finer spatial resolution needed for regional or local-scale analysis. By incorporating the factor change method, we aimed to refine the model output and enhance its suitability for our study area, thus ensuring more accurate and localized projections of precipitation and temperature patterns.

## Results

### Error analysis in R-factor prediction

The statistical evaluation of the model performance using five widely accepted goodness-of-fit metrics is presented in Supplementary Table 2: the coefficient of determination (R²), Nash–Sutcliffe Efficiency (NSE), Percent Bias (PBIAS), Normalized Root Mean Square Error (NRMSE), and the RMSE-observations standard deviation ratio (RSR). These indices offer a comprehensive assessment of model accuracy, bias, and consistency. The R² value of 0.97 indicates a “Very Good” fit between observed and simulated values. This high value suggests that about 97% of the variance in the observed data is successfully explained by the model, highlighting its strong predictive capability. Similarly, the NSE value of 0.96 also falls within the “Very Good” range (NSE > 0.75), reaffirming the model’s effectiveness in capturing the dynamics of the observed time series with minimal deviation. The PBIAS value is 0.12%, which classifies as “Very Good” performance according to the descriptive range provided (± 10% < PBIAS ≤ ± 5%). This metric quantifies the average tendency of the simulated values to be larger or smaller than their observed counterparts. A PBIAS close to zero is ideal, and while this model shows a slight positive bias, it remains within acceptable limits for practical applications. For error-based metrics, the NRMSE is 0.16, which also lies in the “Good” category (0.1 < NRMSE ≤ 0.2). This indicates a moderate level of normalized error, implying that while there is some deviation between observed and predicted values, it is well within tolerable bounds. Lastly, the RSR value of 0.19 places it within the “Very Good” performance bracket (RSR < 0.5), showing that the residual variance is considerably low relative to the standard deviation of observations. Therefore, it could be concluded that the model performs at a “Very Good” to “Good” level across all indices. This robust performance suggests that the model is both reliable and accurate for predictive applications in the studied domain.

### Historical soil erosion analysis

To project future soil erosion in Canadian agricultural lands, we first analyzed historical trends to establish a reference point. An analysis of past data indicated that the average degree of soil erosion induced by runoff was approximately 3.38 t/ha/yr from 1986 to 2016 (https://open.canada.ca/data/en/dataset/b52b3c91-e0eb-47d1-aea5-fa5428254512). Notably, a declining trend was observed, with erosion rates in 2016 being lower than those in 1986. This trend was further supported by the USLE equation results (2009–2020), which estimated an average soil loss of 3.83 t/ha/yr, showing a gradual decrease by 2020.

### Future soil erosion results

**Fig. 4:**
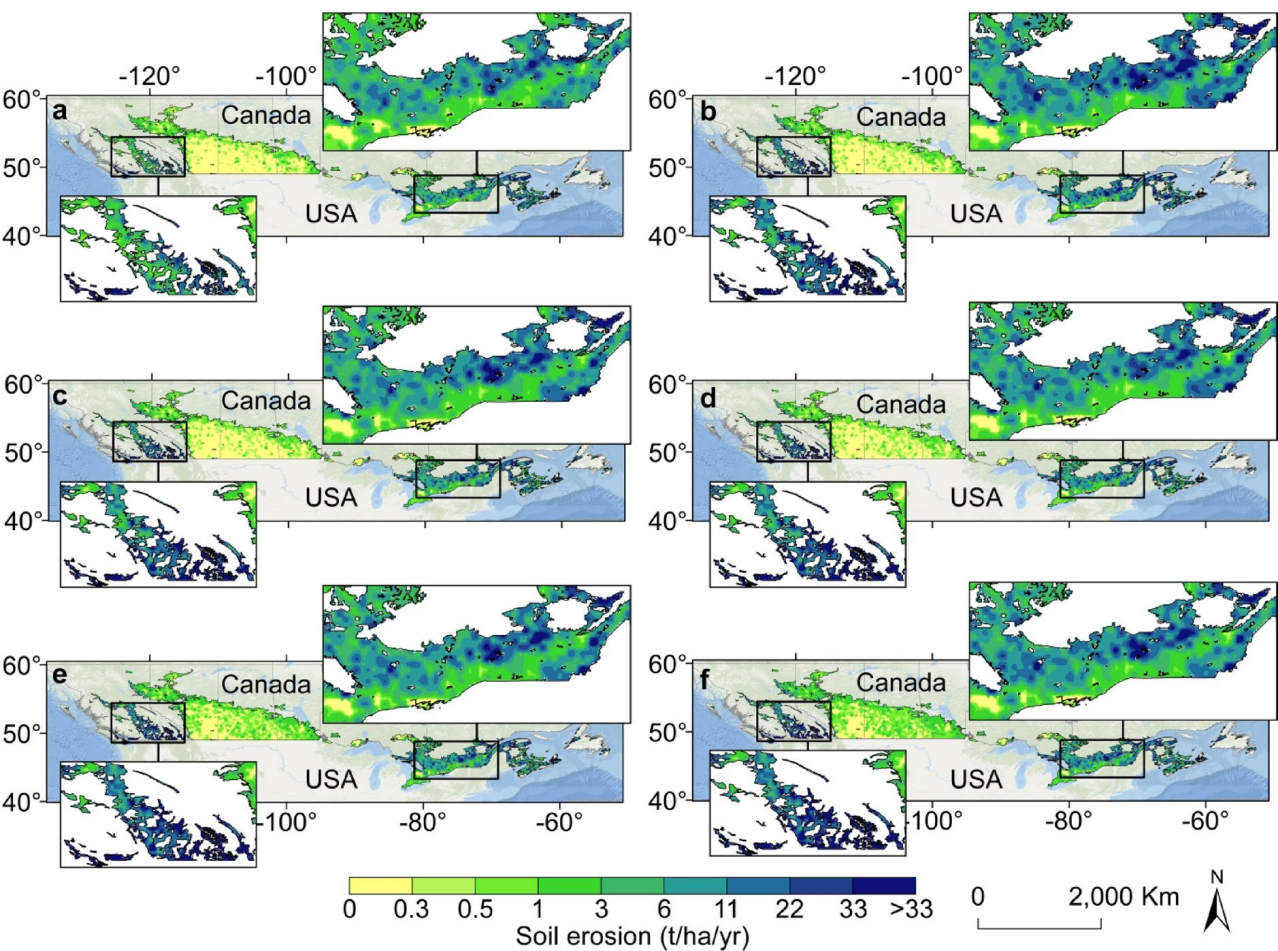
Projected soil erosion from 2076–2100 under the five climate change scenarios. (**a**) Observed soil erosion in 2020. (**b–f**) Predicted soil erosion from 2076–2100 under the (**b**) SSP119, (**c**) SSP126, (**d**) SSP245, (**e**) SSP370, and (**f**) SSP585 scenarios. The map was generated using ArcMap (version 10.8.2), available at https://desktop.arcgis.com.

The projected soil erosion from 2076 to 2100 under five distinct climate change scenarios, which are part of SSP119, SSP126, SSP245, SSP370, and SSP585, is shown in Fig. 4. Analyzing soil erosion in Canadian agricultural lands from 2076 to 2100 under various climate change scenarios provides a nuanced picture of potential erosion risks. From a national average perspective, the data suggest that soil erosion rates are modest, ranging from 4 to 6 tonnes per hectare per year across all scenarios. However, this average value masks significant regional differences that are critical for formulating targeted soil conservation strategies. The national average erosion rates reported in this study were derived from model outputs at a 25 km resolution. While this resolution effectively captures large-scale erosion trends, it may smooth out localized variations that could be more apparent in higher-resolution analyses. The choice of this scale was driven by the need to ensure computational efficiency and compatibility with climate projections, as well as to align with previous large-scale assessments. While finer-resolution modeling (e.g., at 30 m) could provide more detailed insights into localized erosion hotspots, the 25 km scale remains appropriate for identifying broader erosion patterns across the country.

Over 80% of the land remains within the very low erosion category (0–6 t/ha/yr), ensuring relative soil stability. However, 6.5–9% of the area is projected to fall into the low erosion category (6–11 t/ha/yr), whereas 4.7–6.5% could experience moderate erosion (11–22 t/ha/yr). Although high (22–33 t/ha/yr) and severe erosion (> 33 t/ha/yr) rates are less common, their presence, particularly under SSP119, SSP245, and SSP585, highlights the need for targeted conservation strategies.

**Fig. 5:**
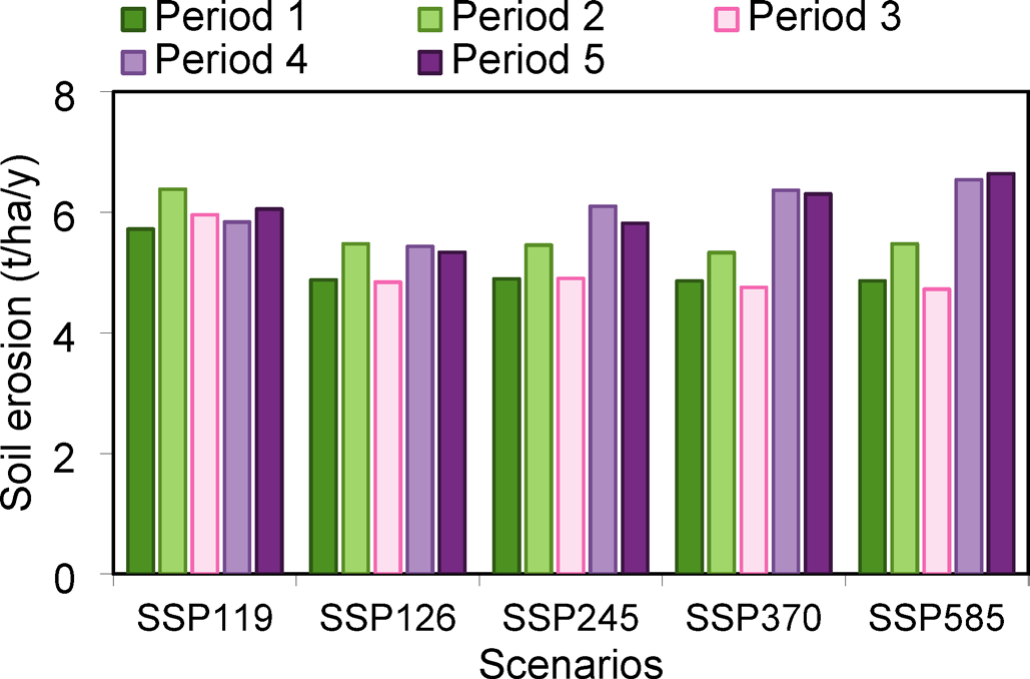
Projected soil erosion (t/ha/yr) in the study area under the various SSPs. Vertical bars show average soil erosion across five time periods (Period 1: 2024–2030, Period 2: 2031–2040, Period 3: 2041–2050, Period 4: 2051–2075, and Period 5: 2076–2100) under different SSP scenarios.

Long-term projections from 2030 to 2100 reveal temporal fluctuations in soil erosion rates under different SSP scenarios (Fig. 5). Under SSP119, soil erosion increased from 5.72 t/ha/yr by 2030 to a peak of 6.38 t/ha/yr by 2040, eventually decreasing to 6.05 t/ha/yr by 2100. Scenario SSP119 is a notable exception in most respects. The soil erosion rate was significantly high in 2040 and 2100, with the remaining periods exhibiting soil loss rates lower than 6 t/ha/yr. Similarly, soil erosion under SSP126, at 5.47 t/ha/yr by 2040, decreased to 5.33 t/ha/yr by 2100. Under SSP245, the projected soil erosion rate increased from 4.89 t/ha/yr by 2030 to a peak of 6.1 t/ha/yr by 2075, followed by a slight decline by 2100 to 5.81 t/ha/yr. Soil erosion under SSP370 and SSP585 exhibited similar trends, increasing from approximately 4.86 t/ha/yr by 2030 to 5.33 and 5.47 t/ha/yr by 2040, respectively, before decreasing to below the starting level in the 2040–2050 period. Soil erosion under these scenarios ultimately reached 6.3 and 6.64 t/ha/yr, respectively, despite a peak of 6.36 t/ha/yr by 2075 under SSP370. These variations underscore the impacts of different climate trajectories on soil degradation, with SSP119 and SSP126 resulting in relatively lower long-term risks, whereas SSP370 and SSP585 resulting in significant erosion increases, emphasizing the necessity of soil conservation strategies.

### Provincial soil erosion changes

**Fig. 6:**
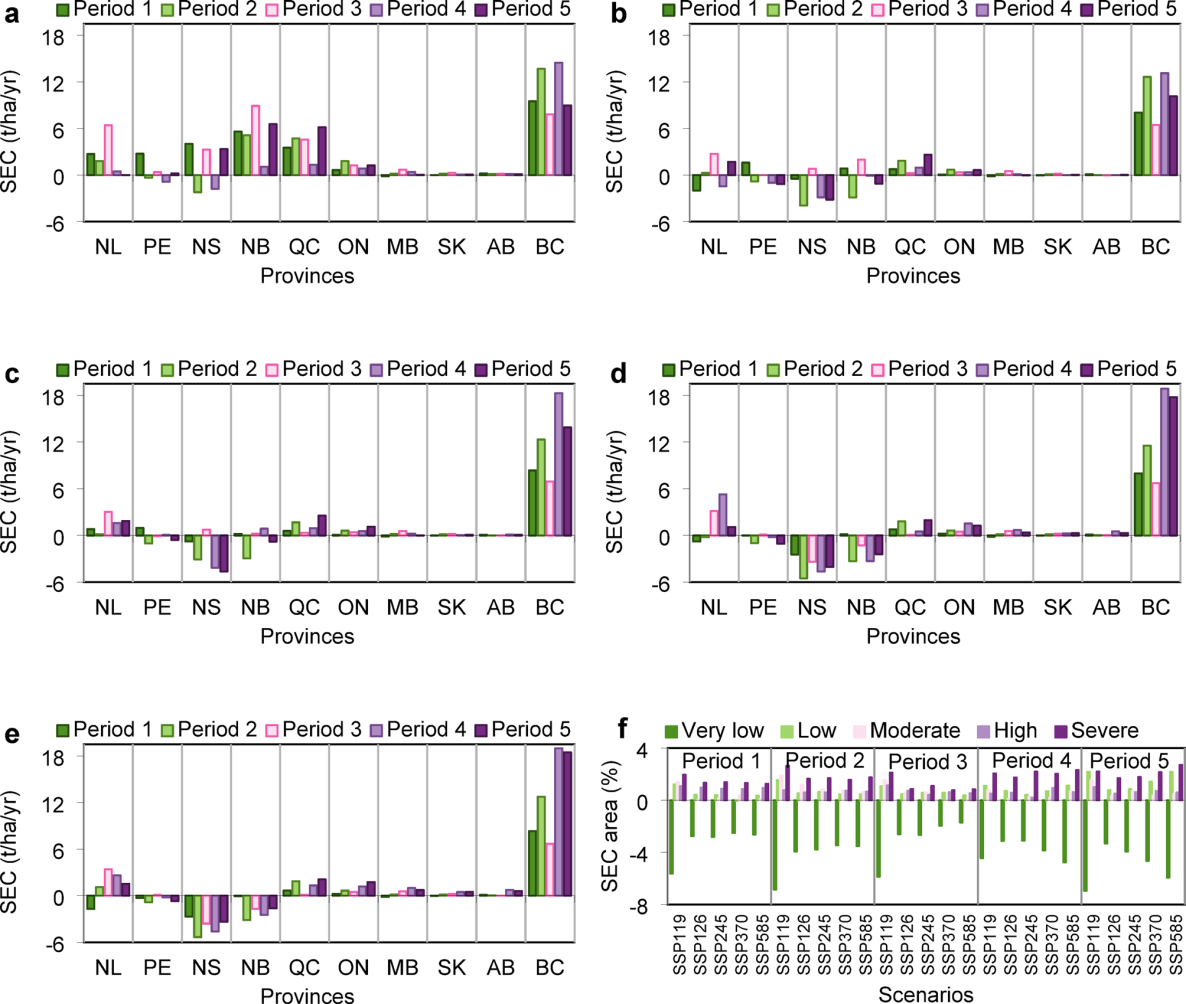
Projected soil erosion during the different periods based on the five climate change scenarios. SEC in provinces relative to soil erosion in 2020 during different periods (Period 1: 2024–2030, Period 2: 2031–2040, Period 3: 2041–2050, Period 4: 2051–2075, Period 5: 2076–2100) via (**a**) SSP119 (**b**) SSP126 (**c**) SSP245 (**d**) SSP370 (**e**) SSP585 (**f**) Percentage of the projected SEC area in the various risk classes under the different climate change scenarios.

The projected soil erosion change (SEC) in provinces during the different periods under the five climate change scenarios is shown in Fig. 6a–e. The suite of charts provided offers a detailed visual representation of soil erosion projections across Canadian agricultural lands under various climate change scenarios during different periods (i.e., 2024–2030, 2031–2040, 2041–2050, 2051–2075, and 2076–2100). These scenarios are part of the SSP scenarios (SSP119, SSP126, SSP245, SSP370, and SSP585) that aim to model different soil erosion rates on the basis of the intensity of climate change and associated mitigation efforts. According to Fig. 6a-e, the SECs in Québec (QC), Ontario (ON), and British Columbia (BC) Provinces increased during the projected periods under all the scenarios compared with those in 2020. The SECs of the QC and ON samples ranged from 0.1 to 6.1 and 0.06–1.7 t/ha/yr, respectively, under the various scenarios. BC exhibited the most significant erosion increase among all provinces, with an average SEC ranging from 6.6 to 18.9 t/ha/yr. Compared with those in 2020, Manitoba (MB), Alberta (AB) and Saskatchewan (SK) presented the lowest rates of erosion change, with SEC rates ranging from − 0.15 to 1 t/ha/yr. Nova Scotia (NS) demonstrated the greatest decrease in the SEC across all the scenarios, particularly under SSP370 and SSP585. The SEC in NS varied between − 5.5 and 4 t/ha/yr. New Brunswick (NB) Province also exhibited a decline in the SEC. Specifically, toward SSP585, a more significant reduction in erosion was observed, and the range fluctuated between − 3.2 and 8.9 t/ha/yr. For Prince Edward Island (PE), the SSP 119 scenario significantly increased erosion, but there was a decreasing trend under the other scenarios. The SEC in PE was relatively low, ranging from − 1.1 to 2.7 t/ha/yr. The SEC in Newfoundland and Labrador (NL) typically increased under most scenarios, but this increase was smaller under the subsequent scenarios, fluctuating between − 1.9 and 6.4 t/ha/yr.

By 2100, high-erosion areas expand, particularly in western Canada, with SSP119 showing the greatest increase in soil erosion due to projected precipitation patterns (Fig. 6f). The main reason for this finding is that this scenario involves an optimistic view of precipitation^43^; as a result, the value of the R factor increases under this scenario, resulting in a larger predicted erosion amount. SSP245 and SSP126 show moderate expansion of high-erosion areas, whereas SSP370 and SSP585 indicate substantial increases by 2075 and 2100. The highest greenhouse gas emission scenario (SSP585) results in the most severe erosion increase, emphasizing the need for robust soil conservation efforts.

All the scenarios indicated an increase in soil erosion over time, which suggests that without significant mitigation efforts, soil degradation will become a more severe problem in the future. The increase in soil erosion is particularly notable in the western regions of Canada, which could be due to a combination of factors such as precipitation patterns, land uses, and soil management practices. The soil erosion intensity differed across the various SSP scenarios, with SSP585 usually resulting in the most severe outcomes. This suggests that the pathway chosen by society (in terms of emissions and development) significantly impacts environmental outcomes such as soil erosion.

### Spatial distribution of areas prone to severe erosion

On the basis of data provided by Canadian agricultural authorities for 2020, the distribution of areas with high and severe soil erosion across the various provinces indicates a significant environmental challenge. The spatial distribution data from 2020 revealed that BC had the highest proportion of agricultural land affected by severe erosion (12.42%), followed by QC (11.07%), NB (8.53%), and NS (8.2%). ON had the lowest impact, with only 1.93% of the agricultural land affected.

**Fig. 7:**
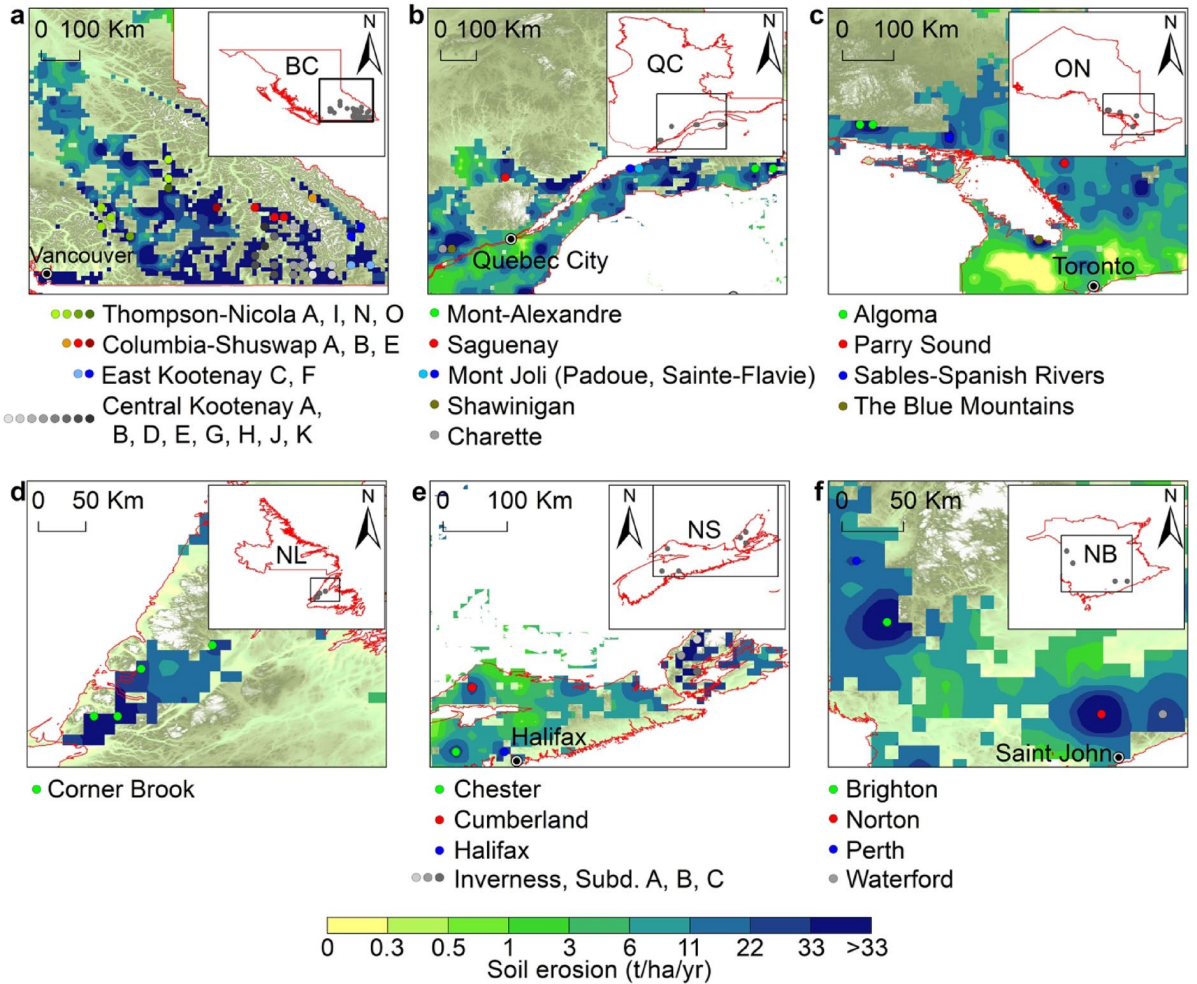
Distribution of regions with high and severe soil erosion risks in Canada. The colored points indicate the centers of pixels with high and severe soil erosion from 2030–2100 under all the scenarios. Each point with a specific color tone denotes a specific area written next to each point. (**a**) High- and severe-risk areas in British Columbia. (**b**) High- and high-risk areas in Quebec. (**c**) High- and high-risk areas in Ontario. (**d**) High- and severe-risk areas in Newfoundland and Labrador. (**e**) High- and severe-risk areas in Nova Scotia. (**f**) High- and severe-risk areas in New Brunswick. The erosion risk layer overlaps the DEM to improve clarity and enhance the topographic context and visualization. The map was generated using ArcMap (version 10.8.2), available at https://desktop.arcgis.com.

With respect to the projected soil erosion in BC, in Central Kootenay regions A, B, D, E, G, H, J, and K, different scenarios—SSP119, SSP126, SSP245, SSP370, and SSP585—have varying degrees of soil erosion, identifying specific locations within these areas that are likely to be impacted. Similarly, under various scenarios, Columbia–Shuswap areas A, B, and E present soil erosion rates exceeding 22 tons/ha/year at numerous locations. East Kootenay regions C and F also presented potential soil erosion issues across multiple scenarios, suggesting that these areas are key targets for conservation efforts. Notably, the Thompson Nicola regions A, I, N and O exhibited consistent projections across all SSP scenarios, indicating significant soil erosion risks (Fig. 7a).

In the QC, numerous regions experienced substantial erosion under all the scenarios. Some of these areas are located in agricultural fields within the northern part of the St. Lawrence River, such as Saguenay, whereas other sections are located mostly between Quebec city and Montreal, including Charette and Shawinigan. Agricultural fields around Mont-Joli in the southern parts of the St. Lawrence River exhibited significant erosion potential. Mont-Alexandre demonstrated notable soil erosion under the different SSP scenarios, indicating geographical variance in the risk of soil erosion (Fig. 7b). The southern parts of Algoma, the Sables-Spanish Rivers, the southern parts of Georgian Bay around the Blue Mountains, and Parry Sound in Ontario experienced severe erosion under all scenarios (Fig. 7c). The lands located southwest of Corner Brook in NL showed possible severe erosion under all the scenarios, emphasizing the diverse effects of the different climate scenarios (Fig. 7d). Similar situations were observed in the NS in the surrounding area of Lake Ainslie (Fig. 7e). Significant erosion is expected in the northern part of Saint-John and the western regions of NB under all the scenarios (Fig. 7f).

This comprehensive summary emphasizes the critical need for focused soil conservation and land management practices in areas identified as most vulnerable and highlights the importance of incorporating a range of climate and development pathways into soil conservation planning efforts to address the anticipated challenges by 2030.

The analysis revealed a clear trend of increasing soil erosion across various regions under the different climate scenarios. Regions such as Central Kootenay and Columbia Shuswap were frequently identified, indicating the need for targeted conservation efforts (Fig. 7a). The consistency among certain identified areas across multiple projection years and scenarios suggests that these regions are particularly vulnerable to soil erosion and should be prioritized for soil conservation and sustainable land management practices. The variation in soil erosion projections under the different SSP scenarios highlights the impacts of different types of climate change and socioeconomic pathways on soil degradation. These findings demonstrate the importance of incorporating climate change mitigation and adaptation strategies into soil and land management policies. These findings emphasize the need for proactive measures to manage and mitigate soil erosion effectively, considering current trends and future projections.

### Temporal analysis of soil erosion

**Fig. 8:**
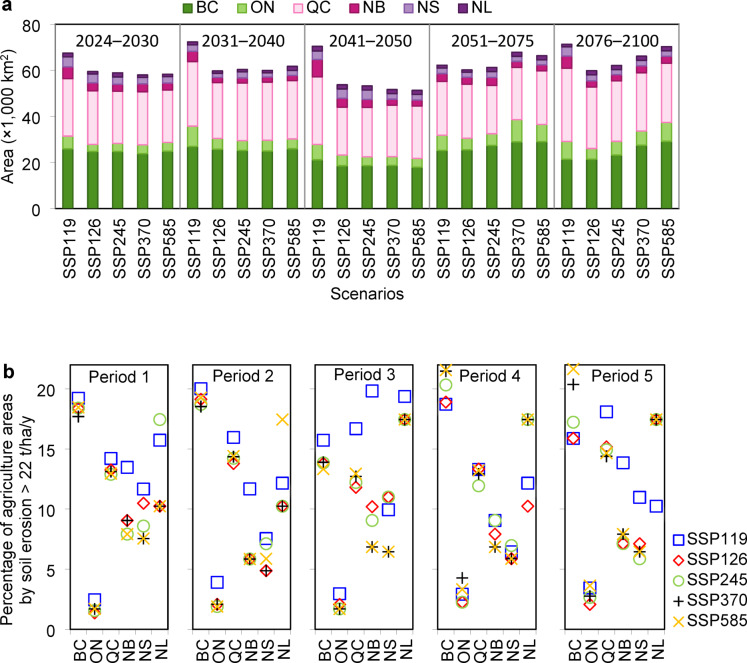
Overview of the soil erosion area characteristics under the various scenarios. **(a)** Agricultural land affected by high and severe soil erosion under the different scenarios from 2030–2100. (**b)** Graphs of the provincial proportions of the agricultural area projections in the high- and severe-risk classes under the various scenarios in different periods (Period 1: 2024–2030; Period 2: 2031–2040; Period 3: 2041–2050; Period 4: 2051–2075; Period 5: 2076–2100).

Temporal analysis of soil erosion revealed that BC and QC have the highest risk of severe erosion in agricultural land from 2030 to 2100 (Fig. 8a). This trend is further illustrated by the provincial proportions of agricultural land in high- and severe-risk classes across different periods (Fig. 8b). In the 2024–2030 period, 17.69–19.22% (23,860–25,916 km²) of BC agricultural lands and 12.88–14.2% (22,632–24,944 km²) of QC agricultural lands faced high or severe erosion risks. By 2040, ON erosion risk will increase, particularly under SSP119, whereas BC and QC erosion risk will continue to increase. By 2050, the BC erosion risk stabilized under SSP585 (13.33% or 17,975 km²), whereas the high-erosion areas of the QC expanded (29,296 km²). By 2075, erosion risks peak in BC and ON under SSP370, with 28,952 km² (20.47%) and 9,612 km² (4.27%) of agricultural lands affected. By 2100, QC and BC remain the most affected, with 31,762 km² and 29,191 km² of high-erosion agricultural land, respectively. The analysis of temporal trends can provide insightful observations regarding the average proportion of locations within each province (BC, QC, and ON) anticipated to face significant soil erosion challenges. From 2040, ON exhibited a notable trend of consistent projected soil erosion levels. With 1.69% and 4.27% of areas exhibiting high soil erosion, the results indicate a sustained risk that warrants continuous monitoring and proactive management strategies to mitigate potential impacts on agriculture and land integrity. In contrast, BC exhibited a greater propensity for high soil erosion, with projected proportions above 13%. Despite this impact, the consistent identification of affected locations in various years suggests an underlying soil erosion issue that requires targeted interventions to prevent localized degradation and preserve ecosystem health. QC is the projected period with a relatively high average proportion of locations at risk (approximately 14.2%) from 2024 to 2030, which indicates significant soil erosion concerns. Intriguingly, there was a notable increase in the projected proportions in subsequent years. This trend might reflect effective soil conservation measures, changes in land use, or climatic conditions that contribute to erosion risk, highlighting the dynamic nature of environmental challenges and the potential for improvement with adequate response measures.

### Sensitivity analysis of time interval selection

While uniform time intervals could simplify certain comparisons, our choice of varying periods is deliberate, scientifically justified, and aligned with the study’s objectives. These intervals were designed to reflect key milestones in climate and socioeconomic projections under Shared Socioeconomic Pathway (SSP) scenarios, capture the non-linear dynamics of soil erosion, and balance the need for detailed near-term insights with broader long-term trends.

To address concerns regarding the use of non-uniform time intervals and their potential to bias comparisons across future periods, we conducted a sensitivity analysis focusing on the rainfall erosivity (R factor) from the USLE. All parameters except the R factor were held constant, and R was recalculated using equal-length intervals (e.g., 15-year periods). Percentage changes were then compared with values from our original intervals. The analysis revealed only minor differences in R values across scenarios, with changes ranging from − 7.8% to + 1.3% (Supplementary Tables 3–7). Notably, even in higher-emission scenarios such as SSP5-8.5, these variations did not reverse the overall trends, affirming the robustness of our results. Therefore, while uniform time windows may offer simplified comparisons, our approach does not introduce systematic bias and remains valid for projecting erosion trajectories within the SSP framework.

## Discussion

The preliminary historical evaluation of soil erosion, which was based on two distinct datasets (i.e., Canadian agricultural data and USLE data), revealed a downward trend in the soil erosion rate. This decreasing trend in soil erosion has crucial implications for agriculture, the environment, and the economy. In Canadian agriculture, the reduction in soil loss suggests enhanced agricultural sustainability, possibly due to improved soil conservation practices, such as reduced tillage or better crop management techniques. Environmentally, this decline bodes well for water quality maintenance, carbon sequestration, and ecosystem restoration. This finding also suggests a reduction in the risk and associated costs of infrastructure damage. The concordant decreasing trend in the USLE data reinforces the findings for Canadian agricultural lands, suggesting that soil conservation efforts are likely effective across different regions and scales. This trend is an encouraging indicator of potential improvements in environmental quality and the sustainability of agricultural practices. Moreover, soil management strategies may contribute to climate change mitigation via increased carbon sequestration. Indeed, both datasets observed decreasing trends in soil erosion induced by runoff signal a positive trajectory toward greater agricultural and environmental resilience. If these trends persist, they could indicate significant benefits across multiple sectors, aligning with sustainable development objectives and increasing the country’s ability to mitigate environmental challenges. To realize these potential benefits, the projected trends in soil erosion were analyzed under the different climate change scenarios.

**Fig. 9:**
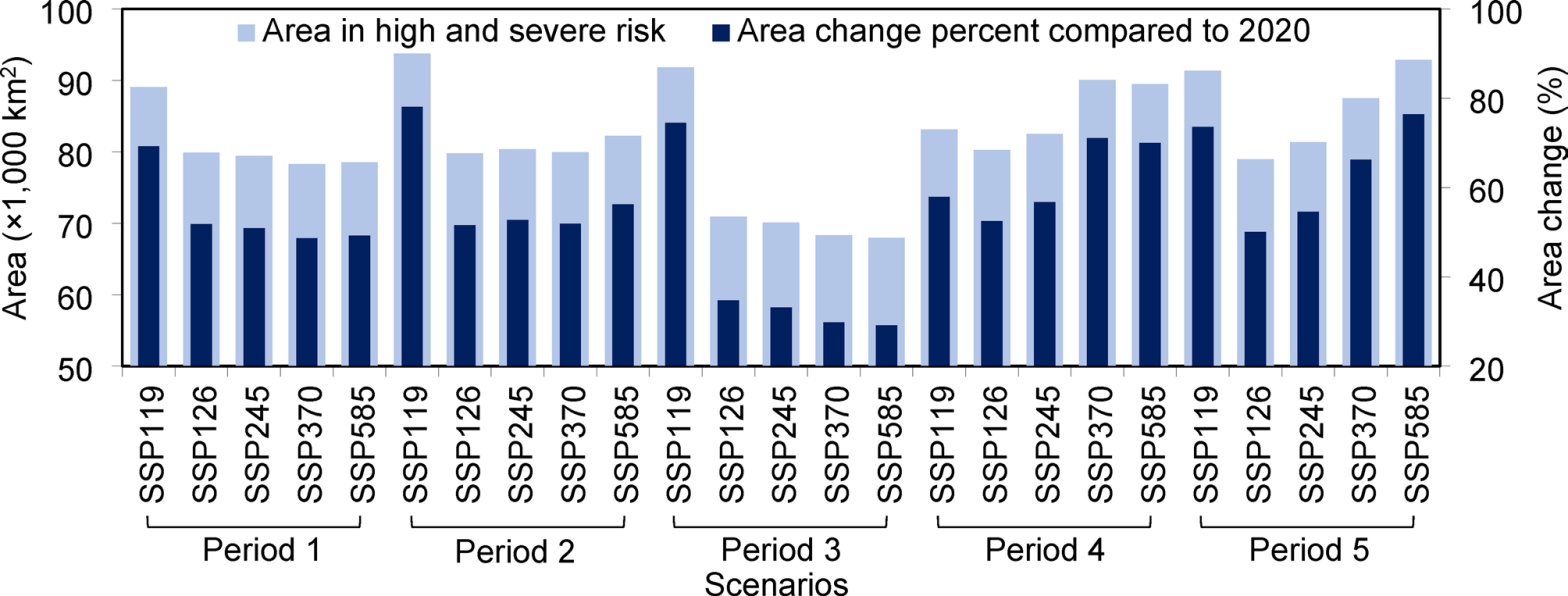
Agricultural lands are projected to present high and severe erosion risks. Agricultural areas in the high and severe soil erosion classes under the different scenarios in different periods (Period 1: 2024–2030; Period 2: 2031–2040; Period 3: 2041–2050; Period 4: 2051–2075; Period 5: 2076–2100). Changes in the high- and severe-risk areas compared with those in 2020 are shown on the secondary y-axis as percentages.

The findings of this study indicate that under various SSP scenarios, Canadian agricultural lands will experience a substantial increase (55.6% on average) in areas at high and severe erosion risk compared with 2020 (Fig. 9). On average, approximately 81,909 km^2^ of these areas will experience high and severe risk. The average increase in high- and severe-risk areas could reach 53.9% by 2030. The predictions indicate that areas with high and severe risk could expand compared with those in 2020, namely, by 58.1%, 40.2%, 61.6%, and 64.1% in 2031–2040, 2041–2050, 2051–2075, and 2076–2100, respectively. The greatest increase in high- and severe-risk areas in agricultural lands was predicted under SSP119 from 2031 to 2040 and SSP585 from 2076 to 2100, with growth rates of 78.1% and 76.3%, respectively. Future risk is strongly influenced by the considered SSPs during the different projected periods^44^.

The numerical consistency across the various SSP scenarios suggests a generalized approach whereby, on average, Canadian agricultural lands will exhibit soil erosion rates close to 5 by 2030. However, the maps indicate that this average does not reflect the spatial variability across the landscape. Erosion is not uniform, with certain regions indicating significantly higher rates, emphasizing the necessity of localized analysis and targeted soil conservation strategies. Notably, the period 2024–2030 represents a critical milestone within the sustainable agriculture strategy of Canada, emphasizing the importance of addressing soil erosion promptly and effectively. The spatial distribution of erosion risk highlights that while national or global averages can guide overarching policy and strategic directions, effective soil conservation must be tailored to the specific needs of each locality. Areas with higher projected erosion risks will require more intensive mitigation efforts, such as implementing contour plowing, strip cropping, terracing, or increased organic matter incorporation. Local factors such as the soil type, topography, climate conditions, land use, and farming practices influence the erosion rate and thus necessitate a region-specific approach. Proactive measures might include promoting conservation agriculture, supporting the development and adoption of erosion-resistant crop varieties, investing in precision farming technologies, and encouraging the adoption of practices such as cover cropping and reduced tillage. The projected consistency in soil erosion rates also presents an opportunity to establish a baseline for the effectiveness of current and future soil conservation initiatives. By monitoring changes in the response to these projections, policymakers and farmers can evaluate the impact of their efforts and adjust strategies accordingly. This localized, data-driven approach is essential for maintaining soil health, ensuring food security, and achieving long-term sustainability in Canadian agriculture. In light of the sustainable agricultural strategy, the need for localized soil conservation measures will increase toward 2030. The success of this strategy will depend on the ability to adaptively manage and implement sustainable agricultural practices that are responsive to the specific erosion risks in each region, ultimately contributing to the resilience and sustainability of the agricultural ecosystem in Canada.

The analysis of the soil erosion projections from 2030 to 2100 across the five distinct climate change scenarios provides crucial insights into the anticipated impacts of climatic shifts on Canadian agricultural lands. These projections, rooted in various SSPs, offer a granular view of the potential increase in the soil erosion rate and the geographical spread of affected areas over time, particularly highlighting the vulnerability of western Canada by 2100. The data revealed a moderate yet steady increase in soil erosion across all the scenarios, with SSP585—associated with the highest greenhouse gas emissions—yielding the greatest erosion rates. This trend demonstrates the critical linkage between climate change trajectories and soil degradation rates, with the western regions of Canada identified as potential hotspots due to factors such as precipitation patterns and land use practices. Such insights are pivotal for directing soil conservation efforts and formulating sustainable land management strategies, especially in high-risk zones.

The SSP scenarios, integrated with Representative Concentration Pathways (RCPs) in frameworks like CMIP6, project socioeconomic and climatic changes over distinct horizons that are not uniformly spaced. Our periods align with these milestones, notably emphasizing 2030 as a critical benchmark outlined in the 2030 Agenda for Sustainable Development and reinforced in assessments like Toward a Sustainable Future: Assessing Progress on the 2030 Sustainable Development Goals. The near-term period (2024–2030) captures progress toward these globally recognized targets, while the subsequent decadal intervals (2031–2040 and 2041–2050) align with mid-century benchmarks commonly used in climate modeling. The longer intervals (2051–2075 and 2076–2100) reflect the stabilization or divergence of SSP trajectories, enabling us to assess cumulative impacts on soil erosion over multi-decadal scales. This structure ensures compatibility with the temporal resolution of climate data and scenario narratives, avoiding artificial truncation of key dynamics.

Our study places greater emphasis on short-term variations, particularly up to 2050, given their relevance to immediate policy and adaptation measures, while acknowledging that uncertainty increases in the latter half of the century^45,46^. Shorter intervals (7 years for 2024–2030 and 10 years for 2031–2040 and 2041–2050) allow us to resolve high-frequency variability in precipitation and erosion drivers, such as intensifying hydrological cycles under SSP scenarios^47^. In contrast, the 25-year periods beyond 2050 are designed to smooth out short-term noise and capture broader, stabilized trends in soil erosion under sustained climate and land-use pressures. This dual-resolution approach reflects the non-linear evolution of erosion processes, ensuring we address both rapid near-term transitions and long-term cumulative effects without overgeneralizing across disparate contexts.

The decision to use non-uniform time intervals is both scientifically grounded and policy-relevant. It aligns with internationally recognized milestones such as the 2030 Agenda and facilitates an accurate representation of non-linear changes in soil erosion. Our sensitivity analysis confirms that this design choice does not significantly affect the interpretation of results. On the contrary, it enables better capture of near-term variability while smoothing out long-term uncertainty, which is essential for designing effective adaptation strategies.

The findings of our study indicate that the projected average soil erosion rate for Canadian agricultural lands from 2024 to 2030 is 5.72 t ha^− 1^ yr^− 1^. This value is remarkably close to the globally measured average erosion rate^48^ of 5.78 t ha^− 1^ yr^− 1^. The similarity between our projection and the observed global average suggests that, despite regional variations, the overall soil erosion dynamics in Canada align with broader global trends. This comparison reinforces the reliability of our model predictions and highlights the importance of proactive soil conservation strategies to mitigate future erosion risks. The results of a recent study^49^ state that a substantial rise in global soil water erosion is anticipated to occur after the mid-21st century. Our findings align with this projection, as our results indicate a continuous increase in soil erosion rates after 2050 across various SSP scenarios. However, while our projections confirm this increasing trend, they also highlight regional variations in erosion intensity, emphasizing the need for localized soil conservation strategies.

While previous studies have focused primarily on global trends, our study provides a region-specific analysis of Canadian agricultural lands, offering new insights into localized erosion risks. Unlike previous global assessments, our study benefits from the use of datasets such as Landsat-based classified land cover data to calculate the C factor, which the Government of Canada has officially categorized. Compared with global-scale estimations, this dataset provides a greater level of accuracy in identifying land cover changes, offering a more precise foundation for soil erosion modeling. The integration of these detailed, region-specific datasets enhances the reliability of our projections and ensures that our findings better reflect the real-world soil erosion dynamics in Canadian agricultural lands. This methodological advantage sets our study apart from previous research and strengthens its applicability in developing targeted soil conservation strategies.

The findings for 2020, provided by Canadian agricultural authorities, offer context for the projections, revealing that over 1.54% of Canadian agricultural lands already present high and severe soil erosion risks. This existing vulnerability, coupled with the projected increase in soil erosion under the various SSP scenarios, calls for an urgent and strategic response to mitigate future risks. Climate change, driven by shifts in temperature and precipitation patterns, amplifies natural hazards such as floods and wildfires^50–52^. These events disrupt vegetation cover, increase surface runoff, and weaken the soil structure, ultimately accelerating soil erosion and land degradation. The analysis emphasized not only the importance of targeted conservation efforts in regions such as Central Kootenay and Columbia Shuswap but also the need for comprehensive soil and land management policies that incorporate climate change mitigation and adaptation strategies. In light of these projections, it becomes vital for policymakers and stakeholders to prioritize the implementation of sustainable land management practices, invest in soil conservation, and integrate climate considerations into agricultural and environmental planning. As such, Canada can safeguard its soil resources, ensure the sustainability of its agricultural and natural ecosystems, and increase its resilience against the increasing challenge of soil erosion under climate change.

## Conclusion

This study comprehensively assesses future soil erosion in Canadian agricultural lands by integrating the USLE model with deep learning and climate change projections. This study highlights the increasing vulnerability of Canadian agricultural lands to soil erosion under future climate change scenarios. While historical trends (1986–2020) indicate a decline in erosion rates, projections for 2024–2100 reveal a 55.6% increase in high- and severe-risk areas, particularly in western Canada, British Columbia, Quebec, and Ontario. These regions require targeted soil conservation measures, such as contour plowing, strip cropping, and reduced tillage, which are tailored to local conditions. The projected acceleration of soil erosion under high-emission scenarios (e.g., SSP585) underscores the urgency of implementing proactive mitigation strategies. To address these challenges effectively, policymakers must integrate adaptive soil conservation practices into national agricultural policies, invest in precision farming technologies, and promote sustainable land management practices. Continuous monitoring and further research are essential for refining predictive models and ensuring long-term agricultural resilience. For future studies, we recommend conducting soil erosion assessments using higher-resolution pixel sizes to improve the spatial accuracy of erosion predictions. Additionally, integrating more advanced soil erosion models alongside the USLE framework could increase the precision of future projections and provide a more comprehensive understanding of erosion dynamics under changing climate conditions.

## Electronic supplementary material

Below is the link to the electronic supplementary material.

Supplementary Material 1

## Data Availability

The datasets employed in this study were sourced from publicly accessible repositories, and all data supporting the findings are duly cited throughout the manuscript.
