# Supplementary material for: Future soil erosion trends in Canadian agricultural lands from runoff and sustainability impacts
Source: Sci Rep. 2025 Jul 2;15:23184. doi: 10.1038/s41598-025-05947-5 (PMC12223118; doi:10.1038/s41598-025-05947-5)
Supplement: Supplementary file 1 — Supplementary Material 1 [file 41598_2025_5947_MOESM1_ESM.docx]

**Supplementary Table 1.** The MATLAB script implements of the LSTM for predicting the rainfall-runoff erosivity factor(R-factor) using lagged precipitation and temperature data combined with spatial coordinate.

| 1 | clc |
| --- | --- |
| 2 | clear |
| 3 | close all |
| 4 |  |
| 5 | %% Data generation |
| 6 | % Pr: Precipitation, Temperature, R-Factor |
| 7 | load Data.mat |
| 8 |  |
| 9 | %% Normalization |
| 10 | [Coordinate_N,~,~] = Normalization(Coordinate); % Normalize spatial coordinates |
| 11 | [Pr_N,~,~] = Normalization(Pr_obs); % Normalize precipitation data |
| 12 | [T_N,~,~] = Normalization(T_obs); % Normalize temperature data |
| 13 | [R_N, minValue, maxValue] = Normalization(R); % Normalize R-factor and retain min/max for denormalization |
| 14 |  |
| 15 | %% Lag definition |
| 16 | Lag = 12; % Number of time lags (months or steps) |
| 17 | j = length(Coordinate); % Number of time series (columns) |
| 18 |  |
| 19 | % Generate lagged versions of normalized data |
| 20 | Pr_N = TS(Lag, j, Pr_N); |
| 21 | R_N = TS(Lag, j, R_N); |
| 22 | T_N = TS(Lag, j, T_N); |
| 23 |  |
| 24 | %% Input combinations |
| 25 | In = [Pr_N(:,[1 2 12]) T_N(:,[1 2 12]) Coordinate_N]; |
| 26 | Out = R_N(:,end); |
| 27 |  |
| 28 | % Split the full dataset into Train (50%), Validation (25%), and Independent Test (25%) |
| 29 | n = size(In, 1); |
| 30 | nTrain = floor(0.5 * n); |
| 31 | nVal = floor(0.25 * n); |
| 32 | nTest = n - nTrain - nVal; |
| 33 |  |
| 34 | trainIdx = 1:nTrain; |
| 35 | valIdx = nTrain+1:nTrain+nVal; |
| 36 | testIdx = nTrain+nVal+1:n; |
| 37 |  |
| 38 | TrainIn = In(trainIdx,:); |
| 39 | TrainOut = Out(trainIdx,:); |
| 40 | ValIn = In(valIdx,:); |
| 41 | ValOut = Out(valIdx,:); |
| 42 | TestIn = In(testIdx,:); |
| 43 | TestOut = Out(testIdx,:); |
| 44 |  |
| 45 | %% Cross-Validation on Training + Validation Set |
| 46 | k = 10; % Number of folds |
| 47 | cv = cvpartition(length(trainIdx) + length(valIdx),'KFold',k); |
| 48 | rmse_list = zeros(k,1); |
| 49 | r2_list = zeros(k,1); |
| 50 |  |
| 51 | allIn = [TrainIn; ValIn]; |
| 52 | allOut = [TrainOut; ValOut]; |
| 53 |  |
| 54 | for fold = 1:k |
| 55 | trainFoldIdx = training(cv, fold); |
| 56 | valFoldIdx = test(cv, fold); |
| 57 |  |
| 58 | TrainingInputs = allIn(trainFoldIdx,:); |
| 59 | TrainingOutputs = allOut(trainFoldIdx,:); |
| 60 | ValidationInputs = allIn(valFoldIdx,:); |
| 61 | ValidationOutputs = allOut(valFoldIdx,:); |
| 62 |  |
| 63 | %% Modeling |
| 64 | numFeatures = size(TrainingInputs,2); |
| 65 | numResponses = 1; |
| 66 | numHiddenUnits= 100; |
| 67 |  |
| 68 | layers = [ ... |
| 69 | sequenceInputLayer(numFeatures) |
| 70 | lstmLayer(numHiddenUnits) |
| 71 | fullyConnectedLayer(numResponses) |
| 72 | regressionLayer]; |
| 73 |  |
| 74 | options = trainingOptions('rmsprop', ... |
| 75 | 'ValidationData',{ValidationInputs,ValidationOutputs}, ... |
| 76 | 'MaxEpochs',2000, ... |
| 77 | 'MiniBatchSize',120, ... |
| 78 | 'GradientThreshold',0.5, ... |
| 79 | 'InitialLearnRate',0.005, ... |
| 80 | 'LearnRateSchedule','piecewise', ... |
| 81 | 'LearnRateDropPeriod',125, ... |
| 82 | 'LearnRateDropFactor',0.9, ... |
| 83 | 'Verbose',0); |
| 84 |  |
| 85 | net = trainNetwork(TrainingInputs,TrainingOutputs,layers,options); |
| 86 |  |
| 87 | %% Prediction for Training, Validation, and Independent Test |
| 88 | PredTrain_N = predict(net, TrainingInputs); |
| 89 | PredVal_N = predict(net, ValidationInputs); |
| 90 | PredTest_N = predict(net, TestIn); |
| 91 |  |
| 92 | PredTrain = Denormalization(PredTrain_N, minValue, maxValue); |
| 93 | PredVal = Denormalization(PredVal_N, minValue, maxValue); |
| 94 | PredTest = Denormalization(PredTest_N, minValue, maxValue); |
| 95 |  |
| 96 | ActualTrain = Denormalization(TrainingOutputs, minValue, maxValue); |
| 97 | ActualVal = Denormalization(ValidationOutputs, minValue, maxValue); |
| 98 | ActualTest = Denormalization(TestOut, minValue, maxValue); |
| 99 |  |
| 100 | % Validation performance only (CV focus) |
| 101 | rmse_list(fold) = sqrt(mean((PredVal - ActualVal).^2)); |
| 102 | r2_list(fold) = 1 - sum((PredVal - ActualVal).^2) / sum((ActualVal - mean(ActualVal)).^2); |
| 103 | end |
| 104 |  |
| 105 | %% Display average performance across folds (on validation set) |
| 106 | mean_rmse = mean(rmse_list); |
| 107 | mean_r2 = mean(r2_list); |
| 108 |  |
| 109 | %% ======================= Supporting Functions ============================ |
| 110 |  |
| 111 | %% Normalization Function |
| 112 | function [normalizedMatrix, minValue, maxValue] = Normalization(dataMatrix) |
| 113 | minValue = min(dataMatrix); |
| 114 | maxValue = max(dataMatrix); |
| 115 | normalizedMatrix = (dataMatrix - minValue) ./ (maxValue - minValue); |
| 116 | end |
| 117 |  |
| 118 | %% Denormalization Function |
| 119 | function denormalizedMatrix = Denormalization(normalizedMatrix, minValue, maxValue) |
| 120 | denormalizedMatrix = normalizedMatrix .* (maxValue - minValue) + minValue; |
| 121 | end |
| 122 |  |
| 123 | %% Lag Generation Function |
| 124 | function combinedMatrix = TS(Lag, j, timeSeriesMatrix) |
| 125 | laggedMatrices = cell(1, j); |
| 126 | for k = 1:j |
| 127 | timeSeries = timeSeriesMatrix(:, k); |
| 128 | laggedMatrix = zeros(length(timeSeriesMatrix) - Lag, Lag + 1); |
| 129 | for i = 1:(length(timeSeriesMatrix) - Lag) |
| 130 | laggedMatrix(i, :) = timeSeries(i:(i + Lag)); |
| 131 | end |
| 132 | laggedMatrices{k} = laggedMatrix; |
| 133 | end |
| 134 | combinedMatrix = vertcat(laggedMatrices{:}); |
| 135 | end |

**Supplementary Table 2.** Classification criteria, performance levels and their values in the current study for five statistical evaluation metrics.

| **References** | **Equation** | **Descriptive performance** | **Value ranges** | **Results of the current study** |
| --- | --- | --- | --- | --- |
| [1-2] | $R^{2}=\left(\frac{\sum_{i=1}^{N} \left(O_{i}-\overline{O}\right) \sum_{i=1}^{N} \left(M_{i}-\overline{M}\right)}{\sqrt{\sum_{i=1}^{N} \left(O_{i}-\overline{O}\right)^{2} \sum_{i=1}^{N} \left(M_{i}-\overline{M}\right)^{2}}}\right)^{2}$ | Unsatisfactory | R^2^ < 0.5 | 0.97 |
|  |  | Satisfactory | 0.5 < R^2^ < 0.6 |  |
|  |  | Good | 0.6 < R^2^ < 0.7 |  |
|  |  | **Very Good** | 0.7 < R^2^ < 1 |  |
| [1-3] | $NSE=1-\frac{\sum_{i=1}^{N} (M_{i}-O_{i})^{2}}{\sum_{i=1}^{N} (M_{i}-\overline{O})^{2}}$ | Unsatisfactory | NSE < 0.4 | 0.96 |
|  |  | Acceptable | 0.4 < NSE < 0.5 |  |
|  |  | Satisfactory | 0.5 < NSE < 0.65 |  |
|  |  | Good | 0.65 < NSE < 0.75 |  |
|  |  | **Very Good** | 0.75 < NSE < 1 |  |
| [1, 4] | $PBIAS=\frac{\sum_{i=1}^{N} (M_{i}-O_{i})}{\sum_{i=1}^{N} O_{i}}\times100$ | Unsatisfactory | PBIAS ≥ ±25% | 0.12% |
|  |  | Satisfactory | ±15% ≥ PBIAS > ±25% |  |
|  |  | Good | ±10% ≥ PBIAS > ±5% |  |
|  |  | **Very Good** | ±5% ≥ PBIAS |  |
| [1,5] | **$NRMSE=\frac{\sqrt{\frac{1}{N}\sum_{i=1}^{N} \left(O_{i}-M_{i}\right)^{2}}}{\sum_{i=1}^{N} O_{i}}$** | Unsatisfactory | 0.3 < NRMSE | 0.16 |
|  |  | Satisfactory | 0.2 < NRMSE< 0.3 |  |
|  |  | **Good** | 0.1 < NRMSE < 0.2 |  |
|  |  | Very Good | NRMSE < 0.1 |  |
| [1-2, 6] | **$RSR\text{ }=\text{ }\frac{\sqrt{\sum_{i=1}^{N} \left(O_{i}-M_{i}\right)^{2}}}{\sqrt{\sum_{i=1}^{N} \left(O_{i}-\overline{O}\right)^{2}}}$** | Unsatisfactory | 0.7 < RSR | 0.19 |
|  |  | Satisfactory | 0.6 < RSR < 0.7 |  |
|  |  | Good | 0.5 < RSR < 0.6 |  |
|  |  | **Very good** | 0 < RSR < 0.5 |  |
| *O*: Observed value; *M*: Modeling result; $\overline{O}$: Average of the observed value | | | |  |

**Supplementary Table 3.** Sensitivity analysis results for SSP1-1.9.

| Original Time Interval | Equal Time Interval | Original R factor | New R factor (Equal Intervals) | Percentage Change |
| --- | --- | --- | --- | --- |
| 2024–2030 | 2024–2038 | 59.81570161 | 60.57056081 | 1.261974992 |
| 2031–2040 | 2039–2053 | 60.79925271 | 60.19753823 | -0.989674126 |
| 2041–2050 | 2054–2068 | 60.34691195 | 60.73333076 | 0.640329053 |
| 2051–2075 | 2069–2083 | 60.34753277 | 60.50379608 | 0.258939028 |
| 2076–2100 | 2084–2098 | 60.8188885 | 60.47072683 | -0.572456489 |

**Supplementary Table 4.** Sensitivity analysis results for SSP1-2.6.

| Original Time Interval | Equal Time Interval | Original R factor | New R factor (Equal Intervals) | Percentage Change |
| --- | --- | --- | --- | --- |
| 2024–2030 | 2024–2038 | 58.12147715 | 58.48732131 | 0.629447464 |
| 2031–2040 | 2039–2053 | 58.42931163 | 56.8046554 | -2.780550011 |
| 2041–2050 | 2054–2068 | 56.55301221 | 56.27801209 | -0.486269637 |
| 2051–2075 | 2069–2083 | 56.14033111 | 56.02835257 | -0.199461832 |
| 2076–2100 | 2084–2098 | 56.00800401 | 55.40194016 | -1.08210221 |

**Supplementary Table 5.** Sensitivity analysis results for SSP2-4.5.

| Original Time Interval | Equal Time Interval | Original R factor | New R factor (Equal Intervals) | Percentage Change |
| --- | --- | --- | --- | --- |
| 2024–2030 | 2024–2038 | 58.78177311 | 59.3361369 | 0.943087894 |
| 2031–2040 | 2039–2053 | 59.25590564 | 56.99504762 | -3.815413839 |
| 2041–2050 | 2054–2068 | 56.89788806 | 55.19114471 | -2.999660293 |
| 2051–2075 | 2069–2083 | 55.18505599 | 54.92882709 | -0.464308481 |
| 2076–2100 | 2084–2098 | 54.35117745 | 53.53342268 | -1.504575997 |

**Supplementary Table 6.** Sensitivity analysis results for SSP3-7.0.

| Original Time Interval | Equal Time Interval | Original R factor | New R factor (Equal Intervals) | Percentage Change |
| --- | --- | --- | --- | --- |
| 2024–2030 | 2024–2038 | 60.42660182 | 60.42075512 | -0.009675716 |
| 2031–2040 | 2039–2053 | 59.82041781 | 57.48827816 | -3.89856798 |
| 2041–2050 | 2054–2068 | 57.44221732 | 53.86649342 | -6.224905773 |
| 2051–2075 | 2069–2083 | 53.80005962 | 52.62883671 | -2.17699185 |
| 2076–2100 | 2084–2098 | 52.78353498 | 52.31039905 | -0.896370296 |

**Supplementary Table 7.** Sensitivity analysis results for SSP5-8.5.

| Original Time Interval | Equal Time Interval | Original R factor | New R factor (Equal Intervals) | Percentage Change |
| --- | --- | --- | --- | --- |
| 2024–2030 | 2024–2038 | 61.06859691 | 60.61226994 | -0.747236697 |
| 2031–2040 | 2039–2053 | 59.73872798 | 57.62483556 | -3.538562839 |
| 2041–2050 | 2054–2068 | 57.51526463 | 53.02543225 | -7.806331786 |
| 2051–2075 | 2069–2083 | 53.16912057 | 52.62913824 | -1.015593874 |
| 2076–2100 | 2084–2098 | 54.59982322 | 54.89252243 | 0.536080868 |

**References**

1. Grégoire, G.; Fortin, J.; Ebtehaj, I.; Bonakdari, H. Forecasting Pesticide Use on Golf Courses by Integration of Deep Learning and Decision Tree Techniques. *Agriculture* **2023**, *13*, 1163. <https://doi.org/10.3390/agriculture13061163>.

2. Moriasi, D.N.; Arnold, J.G.; Van Liew, M.W.; Bingner, R.L.; Harmel, R.D.; Veith, T.L. Model evaluation guidelines for systematic quantification of accuracy in watershed simulations. *Transactions of the ASABE* **2007**, *50*, 885-900. <https://doi.org/10.13031/2013.23153>.

3. Boskidis, I.; Gikas, G.; Sylaios, G.; Tsihrintzis, V. Hydrologic and water quality modeling of lower Nestos river basin. *Water resources management* **2012**, *26*, 3023-3051. <https://doi.org/10.1007/s11269-012-0064-7>.

4. Legates, D.R.; McCabe Jr, G.J. Evaluating the use of “goodness‐of‐fit” measures in hydrologic and hydroclimatic model validation. *Water resources research* **1999**, *35*, 233-241. <https://doi.org/10.1029/1998WR900018>.

5. Mihoub, R.; Chabour, N.; Guermoui, M. Modeling soil temperature based on Gaussian process regression in a semi-arid-climate, case study Ghardaia, Algeria. *Geomechanics and Geophysics for Geo-Energy and Geo-Resources* **2016**, *2*, 397-403. <https://doi.org/10.1007/s40948-016-0033-3>.

6. Ayele, G.T.; Teshale, E.Z.; Yu, B.; Rutherfurd, I.D.; Jeong, J. Streamflow and sediment yield prediction for watershed prioritization in the Upper Blue Nile River Basin, Ethiopia. *Water* **2017**, *9*, 782. <https://doi.org/10.3390/w9100782>.
